# The Impact of Abrupt and Fenceline-Weaning Methods on Cattle Stress Response, Live Weight Gain, and Behaviour

**DOI:** 10.3390/ani14111525

**Published:** 2024-05-22

**Authors:** Sarah E. Mac, Sabrina Lomax, Amanda K. Doughty, Peter C. Thomson, Cameron E. F. Clark

**Affiliations:** 1 Livestock Production and Welfare Group, School of Life and Environmental Sciences, Faculty of Science, The University of Sydney, Camden, NSW 2570, Australia; sabrina.lomax@sydney.edu.au (S.L.); cameron.clark@sydney.edu.au (C.E.F.C.); 2 Allflex Australia Pty Ltd., 33 Neumann Road, Capalaba, QLD 4157, Australia; amanda.doughty@msd.com; 3 Sydney School of Veterinary Science, Faculty of Science, The University of Sydney, Camden, NSW 2570, Australia; peter.thomson@sydney.edu.au

**Keywords:** ear tag sensor, maternal separation, two-step weaning, cortisol, average daily gain

## Abstract

**Simple Summary:**

Two-step weaning using an intermediate separation step of nose-flaps or a fenceline aims to decrease the impact of abrupt weaning for both cows and calves, but the previous evaluations of such weaning methods have been conducted over the first step of weaning (4–7 days post-weaning). Additionally, behavioural measurements were collected using interval visual observations. However, the use of sensor technology allows for continuous and objective behavioural measurements. Here, the impact of abrupt and fenceline weaning methods on cattle stress response, live weight gain, and behaviour (using sensor and visual observations) was determined over 14 days. Although both groups were stressed in response to weaning, abruptly weaned animals were more stressed. In general, the impact of stress for fenceline-weaned animals occurred after 3 days of separation by a fenceline. Together, our results suggest that calves be fenceline-weaned for three days followed by total separation.

**Abstract:**

The impact of abrupt (AB) and fenceline (FL) weaning methods on cattle stress response, live weight gain, and behaviour were determined across 14 days. Thirty-two cow–calf pairs were fitted with ear tag sensors (to continuously record behaviour) and allocated to two weaning treatments. After separation, FL calves were maintained in a pen adjacent to the FL cow paddock. The AB calves were transported to a pen removing all contact with the cows. After 7 d, FL cows were transported away from all calf pens. Body weights and salivary samples were collected for all animals on experimental days 0, 7, and 14. Fenceline-weaned calves had a greater duration of rest and rumination with reduced high activity across the first 3 days after separation as compared to abruptly weaned calves in line with the greater occurrences of pacing observed for AB calves. Fenceline-separated cows had greater levels of rest across the first 7 days but similar levels of rumination compared to abruptly separated cows. Fenceline-separated cow activity levels tended to be greater and eating levels were similar across the first three days. Body weight (BW) and cortisol concentrations were similar for AB and FL cattle, but FL cows had lower overall weight gain than the abrupt cows likely due to reduced eating time on days four to seven. Together, these results suggest that calves be fenceline-weaned for three days followed by total separation.

## 1. Introduction

Weaning typically occurs when beef calves are 6 to 8 months of age, achieved through the abrupt cessation of milk consumption from the dam by separation [1,2,3]. Current weaning methods occur earlier than natural dry weaning of beef cows [4]. Early weaning is a necessary husbandry practice to prepare the cow for subsequent parturition and the succeeding lactation by maintaining or increasing its body condition [3,4,5]. The objective of post-weaning management of calves is to either reach liveweight targets for joining as a replacement heifer [6] or to sell these calves into the beef supply chain. However, conventional abrupt weaning typically results in an intense immediate physiological and behavioural stress response in cows and calves, observed by increasing vocalisations and pacing while decreasing eating and rumination duration [7,8]. Greater cortisol concentrations in saliva, faeces, and blood have also been reported for abrupt weaning when compared to two-step weaning [9], but behavioural observations and cortisol collection have been limited to acute changes during the 4 to 7 days following weaning separation [8,9,10,11]. This time period occurs only during the first step of the two-step process. Thus, increasing the time in which both physiological and behavioural data are collected will provide a holistic understanding of the full impact of two-step weaning. 

The stress of abrupt weaning led to recent research investigating alternative weaning methods such as staging the separation processes by first ceasing milk access and then contact between the cow and calf [7,8,12]. The aim of this two-step weaning process was to provide limited visual, auditory, and tactile stimuli between cows and calves while preventing suckling using a fenceline or nose flaps before full separation. Work investigating two-step weaning has shown greater calf weight gain [13,14], no difference in weight gain [9,10,11], and another reported reduced gain [8] when compared with abruptly weaned calves. There are consistent reports of similar total cortisol concentrations between calves weaned abruptly or in two stages [11,15,16], some studies have reported lower cortisol concentrations for fenceline-weaned calves during the first few days after separation [11,15]. Similar to behaviour measurements, cortisol concentration measurements of current research on two-step weaning are limited to the first few days following separation [9,15,16]. As such, there is a paucity of information on the long-term impact of the weaning methods on cow and calf physiology and behaviour with no clear recommendations on the appropriateness of either method for industry. Also, direct visual observations are limited due to differences in inter-observer interpretations, visibility, disturbance of natural behaviour, and the ability to maintain continuous observations for extended durations. Thus, having an objective and continuous form of measurement would provide more robust data in order to provide industry recommendations. 

Precision livestock technology (PLT) allows for automatic, objective measurements whilst increasing labour efficiency, welfare, and production [17]. Such technologies have been used in dairy steer calves for remote health monitoring [18], feed bunk monitoring systems [19], in vivo meat tissue analysis [20,21] and animal-mounted sensors to measure individual behaviours [22]. Sensor technology is being used to enable management decisions through monitoring for heat detection for breeding [23], calving detection [24,25], and heat stress in feedlots [26]. Accelerometer-based ear tag PLT i used to monitor rumination [19,22,27,28], eating [19,27,28], grazing [22], and resting [19,27], providing frequent information to track changes in individual animals. Through PLT, it is now possible to collect objective, continuous data for longer periods of time compared to visual observations. Such technologies now allow for a more thorough assessment of the behavioural impact of differing weaning strategies in order to minimise post-weaning stress response.

Our objectives were to determine the impact of AB and FL weaning methods on cattle stress response through saliva cortisol concentrations, live weight gain, and behaviour, and continuous and visual behavioural measurements over a 14 d period. The hypothesis was that AB cows and calves would display more distressed behaviour, have greater cortisol concentrations, and lower average daily gain when compared to their fenceline treatment counterparts during the first week of separation. 

## 2. Materials and Methods

This experiment was conducted at The University of Sydney commercial sheep and beef property in the Southern Tablelands of NSW, Australia, between April and May 2019 with animal usage approved by the University of Sydney Animal Ethics Committee (Protocol 2018/1401). 

### 2.1. Animals 

Thirty-two Angus cow–calf pairs were enrolled in the experiment. The cows were multiparous, pregnant, and of the same age. Before the experiment commenced, cattle were offered a daily ration of ad libitum oaten hay (8–9 MJ of ME) and 1.5 kg per head of wheat or barley grain. The calves in this experiment ranged between 6 and 8 months of age. Three weeks before separation, the cows and calves were fitted with an accelerometer-based ear tag sensor (Allflex^®^ eSense™, SCR Engineers Ltd., Netanya, Israel), optimised for use in mature dairy cows. These were deployed in the inner part of the left ear between the two cartilage ribs, as per the manufacturer’s installation procedure (Allflex Livestock Intelligence, 2020, Netanya, Israel). Baseline behavioural data were collected from −13 to 0 d from weaning. On the day before weaning (0 d), the cows and calves were separated, weighed using livestock scales (Thunderbird T30/2000, Mudgee, Australia), restrained in a cattle crush, and marked with a numerical visual identification number on either side of their body using tail paint, and had their saliva samples collected while restrained in the crush. A rope halter was used to minimise head movement to allow for safe collection. The liveweight and saliva samples were recorded 0 d, 7 d, and 14 d from weaning. The calves were withheld from the cows for a period of 4 h during baseline collection before reuniting them to allow for accurate cow–calf pairing. After the baseline measurements were collected, the cows were maintained in a set of yards while the calves were individually reintroduced to the cows to confirm the cow–calf pairs (determined by the calf seeking and suckling the cow), and their identification numbers were recorded.

### 2.2. Experimental Design

The cow–calf pairs were allocated to one of two weaning method groups (*n* = 16 pairs/group), (1) fenceline (FL), where cows and calves were separated by a single fence for the first 7 d then fully separated, and (2) abrupt (AB) where cows and calves were fully separated at weaning. The groups were balanced for calf age, weight, and sex. 

#### 2.2.1. Weaning Separation Part 1

On the day of separation, calves were drafted from the cows and sorted into their allocated groups. Abrupt calves were then transported to a holding pen (33 m × 40 m) located approximately 2 km away from the FL pen, and cow paddocks were installed to prevent visual, auditory, and tactile stimuli between the AB cows and calves. Abrupt cows were relocated to a paddock ≥2 km from all the calves. The fenceline calves were located in a pen (15 m × 28 m) directly adjacent to the paddock where the FL cows were maintained and separated by a single fenceline allowing for the cessation of suckling/lactation, while still allowing for limited visual, tactile, and auditory stimuli through the fence [8]. All the calves were offered ad libitum water, oaten hay (8–9 MJ of ME) in hay feeders, wheat or barley grain, and supplementary molasses lick blocks (Olssons calcium mineral block). 

#### 2.2.2. Weaning Separation Part 2

On day 7 of the experiment, the cows and calves were weighed and their saliva samples were collected in their respective groups. Immediately after sample collection, the FL cows were transported to the AB cow paddock ≥2 km away from all the calves to prevent any auditory stimuli and were kept together for the remainder of the experiment. On day 14, the cattle were weighed, their saliva samples were collected, and their ear tags were removed. Upon conclusion of the experiment, all the calves were transported to a weaner paddock while the cows were maintained as a group in a separate grazing paddock. 

### 2.3. Behaviour Data 

#### 2.3.1. Sensor-Derived Behaviour Data

The behaviour of the cattle was recorded automatically via accelerometer-based ear tags (as described in Table 1). Individual cattle sensor-derived data were transmitted to a base station for categorisation of animal behaviour in single-minute resolution according to a proprietary algorithm. These ear tag sensors recorded resting, rumination, activity, and eating/grazing duration [26]. Minutes with no specific behaviour patterns were classified as undefined. The data were categorised into three time periods: baseline (−13 d to 0 d), part 1 separation (1 d to 7 d), and part 2 separation (8 d to 14 d).

#### 2.3.2. Human-Derived Behaviour Data

A visual collection of detailed behaviours was included to further understand the impact of weaning on cows and calves. Focal behaviours for visual observations are described in the ethogram (Table 2). Behaviour was continuously recorded post-weaning using CCTV cameras (NVW-490, Swann Security, Melbourne, Australia) which were positioned in both the AB and FL calf pens. The camera for the FL group was positioned perpendicular to the fenceline to monitor FL cow behaviour (1 d to 7 d) and FL calf behaviour (1 d to 14 d). The abrupt calves were monitored (1 d to 14 d) with the camera positioned facing the pen at the bottom left corner (the direction they were transported from). Behaviour was recorded at the group level as the individual ID of cattle could not be accurately seen at all times, as numbers were often obscured, particularly at night. After the experiment concluded, the CCTV video recordings were downloaded for visual observation of behaviour from the Swann camera system (NVW-490, Swann Security, Melbourne, Australia). Video data were analysed using BORIS (Edition 7.8, Torino, Italy) for the first 15 min of each hour timepoint in block intervals which varied across days as described in Table 3. The frequency of the timepoints was modified from Loberg et al. [9], and the interval duration was modified from Haley et al. [1]. All behaviours were documented as point behaviours to evaluate frequency across time. Close-to-barrier and pacing behaviours were also analysed for the change in total number of calves displaying these behaviours across time. Social and suckling attempt behaviours were not included for the AB calf group as they had no contact with the cows. Due to the limitation of the use of CCTV cameras for monitoring cows in a paddock, no CCTV cameras were used to monitor any cow behaviour after full separation.

### 2.4. Salivary Cortisol Procedure

Whilst restrained in a crush, a rope halter was placed around the cow or calf’s head for further restraint, and a sterile soft plastic 1 mL bulb pipette was inserted between the cheek and lower jaw to access the salivary gland using the animal ethics committee standard operating procedure (Supplementary Material Appendix S1). Saliva was drawn into the pipette, withdrawn from the mouth, and the 1–2 mL sample was expelled into a 5 mL Eppendorf tube and stored in a freezer (≤−20 °C) until processed for cortisol concentration. Upon processing, the samples were thawed at room temperature and centrifuged at 5000 rpm for 15 min at 4 °C. A corticosterone enzyme immunoassay kit (K003-H1W Cortisol ELISA kit, Bio Scientific Pty. Ltd., Kirrawee, Australia) ran through the ASSAYZAP Universal Assay Calculator (Biosoft, Cambridge, UK) and was used to analyse samples with a dilution of 1:4 sample-to-buffer ratio. 

### 2.5. Statistical Analysis

Statistical analyses were conducted in RStudio© (v4.2.5019, Boston, MA, USA) [30], an integrated development environment for R (v4.1.1, Boston, MA, USA) [31]. Cows and calves were analysed separately and between groups for all analyses.

#### 2.5.1. Sensor Behaviour

All sensor behavioural data are represented as days across three time periods: baseline (−10 to 0 d before separation), part 1 separation (day of separation, 1 to 7 d with FL cow–calf pairs sharing the fenceline), and part 2 separation (8 to 14 d with removal of FL cows).

##### Daily Sensor Behaviour

Of the 32 cow–calf pairs, ear tag behaviour data on 30 cows and 31 calves were recorded on a minute basis and summed by day of experiment for a total of 21 days. Cow and calf data were analysed separately. Data were expressed as the proportion (relative frequency) of time each day that each cow or calf was classified as being in each behaviour state. This was calculated separately for each experiment day broken down by AB vs. FL treatment group. For each behaviour, the proportion of a day in the specific behaviour was analysed using a linear mixed model (LMM) using the “lme4” package [32]. These proportions were log-transformed to meet modelling assumptions. Fixed effects of the model were treatment (AB vs. FL), Day, and their interaction (to allow for a different shaped time course for each treatment). Cow ID or Calf ID was the random effect in the model. The emmeans package in R [33] was used to calculate model-based mean proportions, and pairwise comparisons of treatment means on each experiment day were conducted using the “cld” function in the multcomp package [34] and the results were visualised using the ggplot2 [35] package in R.

##### Sensor Behaviour Diversity

From the previous analysis, the proportion of time spent in each behaviour state has been determined for each animal on each day. Then, from these state proportions (*p*), the Shannon diversity index (*H*) was calculated as

$$H=-\sum_{i=1}^{s}p_{i}\log_{e}p_{i}$$

where *s* is the number of behaviour states. Values of *H* increase with an increasing number of behaviour states exhibited but also with more even allocation of time across the different states. It may be considered as a measure of variability for categorical data, like the role of the standard deviation for quantitative data. The maximum diversity occurs when all *p_i_* are equal, i.e., all equal to 1/*s*, and this results in *H*_max_ = log*_e_s*.

The values of behavioural diversity *H* were then analysed with a linear mixed model with the fixed effects of Treatment (AB vs. FL), Day, and their interaction. Cow ID or Calf ID was the random effect in the model [33]. Model-base means were obtained using the emmeans package and the pairwise comparisons of treatment means on each experiment day was conducted using the “cld” and the results were visualised using the ggplot2 package in R.

##### Sensor Behaviour Run Length

Intervals of time (minutes) between changes in behaviour states, also known as run lengths, were calculated after combining all the daily data files. A half minute was added to each observation, due to the potential a behaviour was recorded between the minutes such that it would not be the true record. For example, if a behaviour was recorded for 30 s within a minute, the sensor would display it as 0 min performing this behaviour. This was done separately for cow and calf data. Data manipulation was conducted using the statistical package R.

For each cow or calf, the length of time in the specific behaviour was analysed using a linear mixed model with the “lme4” package. These time intervals were log-transformed to meet modelling assumptions. The fixed effects of the model were treatment (AB vs. FL), Day, and their interaction (to allow for a different shaped time course for each treatment). Cow ID or Calf ID was the random effect in the model. The pairwise comparisons of treatment means on each experiment day were conducted using the “cld” function and results visualised using ggplot2.

#### 2.5.2. Human-Derived Behaviour Observations

Specific visually observed behavioural data were classified into one of two types, namely, count and point. Count refers to the number of animals observed undertaking the specific behaviour at a point of time, and these were noted whenever a change in the count was observed. Behaviours of this type included the number of animals close to the barrier, and the number of animals pacing. Point refers to a behaviour that occurred at a specific point of time, including vocalisation, head out, social and suckling attempt, although the latter was not analysed due to insufficient records. Behaviour mean number is provided for each day with the exception of days 5, 6, and 7 for AB calves due to technological issues.

The count data were analysed using a Poisson GLM. For the calf data, the model fitted was

log*_e_*μ = constant + Treatment + Day + Treatment × Day + log*_e_n*

where μ is the model-based mean count of the behaviours, Treatment is the effect of AB vs. FL weaning separation, Day is the effect of experiment day, Treatment × Day is the interaction term (to allow for different time courses of the two separation methods) and *n* is the number of observations, with log*_e_n* being an offset term in the GLM. The models were fitted using the “glm” function in R, specifying a ‘quasipoisson’ family to allow for under-dispersion of the count data. The model-based means were obtained using the emmeans package [33], and comparison of the two separation methods at each experiment day were obtained using the “cld” function in the multcomp package. For the cow data (FL only), Treatment and Treatment × Day terms were omitted from the model.

The outcome of the point data analyses was the total number of behaviours recorded over the 15 min scan period. These counts tended to be highly over-dispersed, so a negative binomial model was used for the analysis of these data.

log*_e_*μ = constant + Treatment + Day + Treatment × Day

The models were fitted using the “glm.nb” function in the MASS package [36] in R, with use of the emmeans and multcomp packages as above. Again, the Treatment and Treatment × Day terms were omitted from the models for the cow data.

#### 2.5.3. Cortisol Concentrations

For each cow or calf, the cortisol concentrations were analysed using a linear mixed model with the “lme4” package. These time intervals were log-transformed to meet modelling assumptions. The fixed effects of the model were treatment (AB vs. FL), week, and Cow ID or Calf ID was the random effect in the model. All the model-based means for the fixed effects were obtained using the “emmeans” package [33]. The pairwise comparisons of treatment means on each experiment day was conducted using the “cld” function.

#### 2.5.4. Weight and Average Daily Gain

Cow and calf weight were analysed using two linear mixed models using the “lme4” package with weight as the response variables. Within these models, the fixed effects included treatment and week with Cow ID or Calf ID as the random effect. All the model-based means for the fixed effects were obtained using the “emmeans” package [33] and the pairwise comparisons of treatment means for each week was conducted using the “cld” function.in the multcomp package. Cow and calf average daily gain (ADG) were calculated using a contrast function using the weight data. Part 1 and 2 separation all the model-based means for the fixed effects were obtained using the contrast function.

## 3. Results

### 3.1. Sensor Behaviour Data

#### 3.1.1. Daily Sensor Behaviour

**Figure 1:**
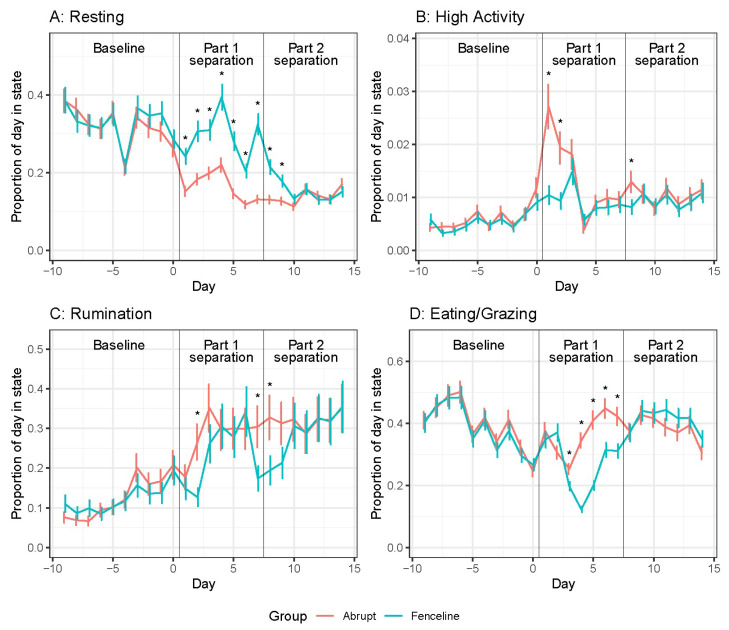
Daily cow behaviour means ± SE representing the proportion of time in that activity. (**A**) Resting, (**B**) High activity, (**C**) Rumination, and (**D**) Eating/Grazing. Pairwise significant differences are indicated with an asterisk (*p* < 0.05).

Mean proportions of time resting, high activity, rumination, and eating for cows are shown in Figure 1. Overall, there was a significant Treatment × Day interaction (see Table S1). The behaviour duration was similar between both groups of cows during the baseline period. During part 1 separation, the AB cows decreased their resting time and remained below baseline for the remainder of the experiment. The fenceline cows rested 10% more from day 1 of separation until day 10 (during part 2 separation) when compared to the AB cows. Both the groups displayed high-activity time immediately after separation. For the first 2 d following separation, the AB cows displayed three times greater levels of high activity than the FL cows. The peak of high activity for the FL cows was on day 3 with days 1 and 2 having similar high activity to baseline. Both the groups increased in rumination after separation; however, the FL cows had less rumination time 2 d after separation, with the AB cows ruminating two times more than the FL cows. Fenceline cow eating time decreased from baseline on days 3 to 7 following separation before returning to baseline whereas AB cow eating time was similar to baseline levels for the entirety of the experiment.

At part 2 separation (day 8), the FL cow resting time decreased, similar to the AB cows. Abrupt cows had another spike in high activity on day 8; however’ this was 2.5 times smaller than the peak on days 1 and 2. A second peak of high activity was not recorded for the FL cows. The abrupt cows ruminated 10% more than the FL cows on days 7 and 8 due to a decrease in FL cow rumination behaviour. The eating time was similar for both groups and was slightly higher than the baseline period.

**Figure 2:**
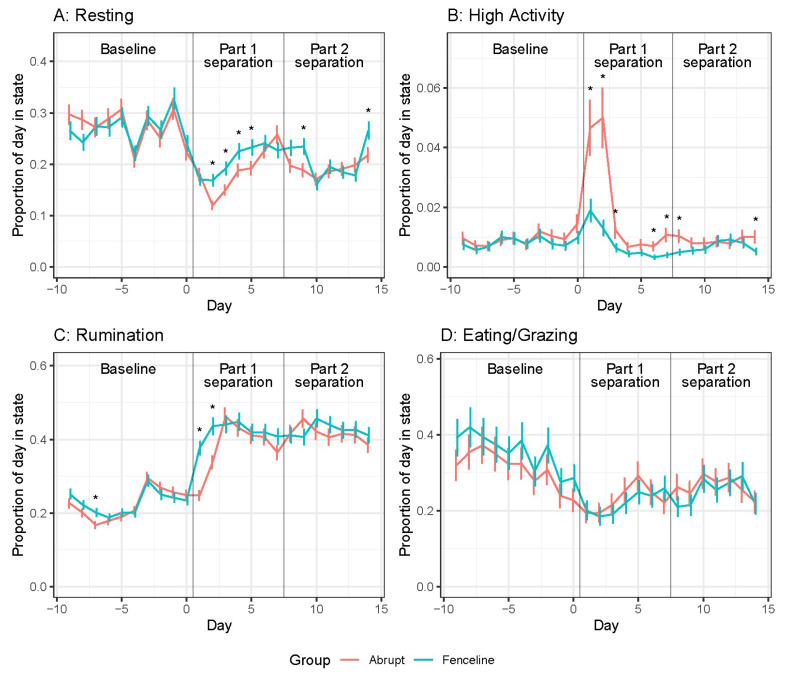
Daily calf behaviour means ± SE representing the proportion of time in that activity. (**A**) Resting, (**B**) High activity, (**C**) Rumination, and (**D**) Eating/Grazing. Pairwise significant differences are indicated with an asterisk (*p* < 0.05).

Calf mean resting, high activity, and rumination times are shown in Figure 2. There were significant Treatment × Day interactions for resting, rumination, and eating/grazing (see Table S2) but not for high activity (*p* = 0.73). During part 1 separation, calf resting time decreased for both groups over the first two days after separation compared to baseline levels. During this time, the FL calves had greater resting and rumination times. Although both groups of calves had a surge of high activity on day 1 and 2, the AB calves increased three times their baseline on day 1 compared to the FL increasing two times their baseline. Both groups showed increased rumination after separation; however, the FL calves spent a greater proportion of time, spending 13% and 10% more time ruminating for the first 2 d directly after separation while remaining similar thereafter. Their eating times were similar for the entirety of the experiment and decreased slightly after separation.

During separation part 2, both groups’ resting times remained below baseline and were similar every day except on days 9 and 14 when the FL calves had greater resting time. The abrupt calves had another increase in high activity from days 6 to 8 that was four times less than the initial peak at day 1. There was no increase in high activity for the FL calves during part 2 separation. Rumination was similar between the groups during part 2 separation and remained greater than baseline and similar to part 1 separation.

#### 3.1.2. Sensor Behaviour Diversity

**Figure 3:**
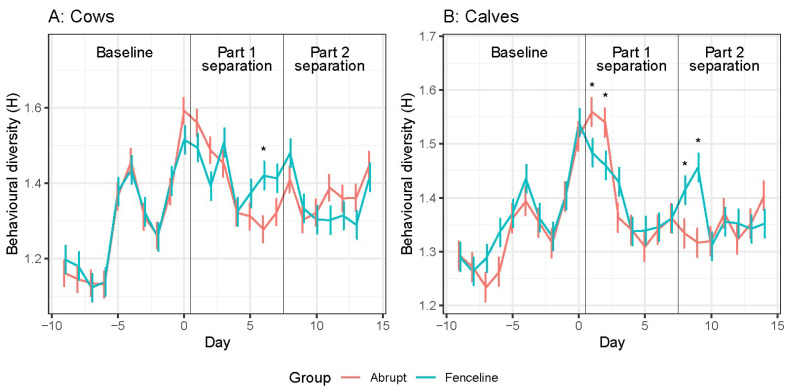
Daily cow (**A**) and calf (**B**) behaviour diversity value (H) means ± SE. Pairwise significant differences are indicated with an asterisk (*p* < 0.05).

Mean cow behaviour diversity is shown in Figure 3A. Cow behavioural diversity increased during the baseline period and then reduced after separation, for both the treatment groups. The greatest behavioural diversity for the cows occurred on day 1. However, on day 6, the FL cows had less behavioural diversity compared to the AB cows. There was a significant Time × Treatment interaction (see Table S3) with a tendency for the AB cows to have greater diversity than the FL cows during part 1 separation, from day 1 to 4, with a significant increase in FL cow behaviour diversity on day 5. Both groups had an increase in behavioural diversity on day 8.

Mean calf behavioural diversity is shown in Figure 3B. There was a significant Time × Treatment interaction (see Table S3). The calves followed a similar pattern of behavioural diversity as the cows during baseline and declined in diversity after separation. On days 1 and 2, the AB calves had greater behavioural diversity than the FL calves. On day 3, calf behaviour remained consistent for the remainder of part 1 separation. During part 2 separation, behavioural diversity was similar between the groups every day except days 8 and 9 when the FL calves had greater diversity.

#### 3.1.3. Sensor Run Length

**Figure 4:**
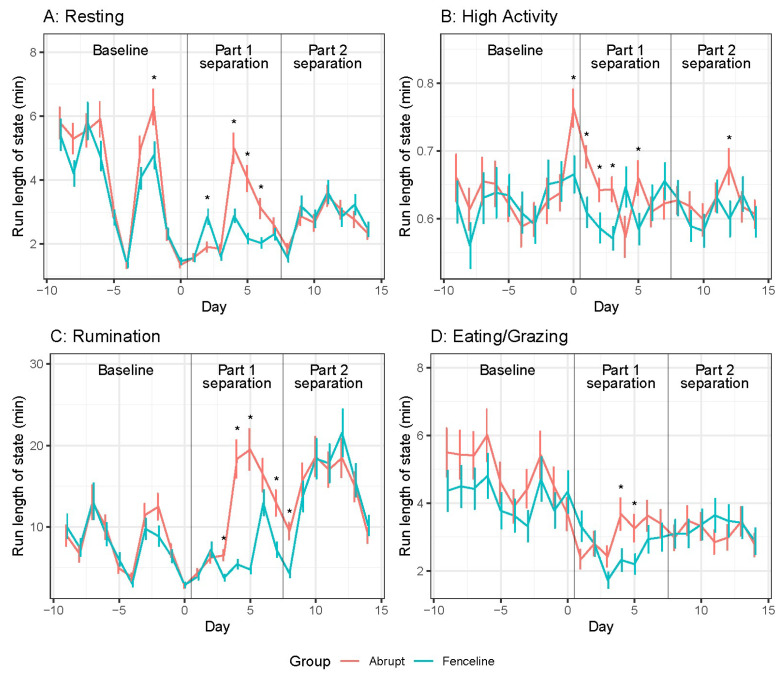
Daily cow behaviour run length means ± SE. (**A**) Resting (**B**) High activity, (**C**) Rumination, and (**D**) Eating/Grazing. Pairwise significant differences are indicated with an asterisk (*p* < 0.05).

Cow mean resting, high activity, and rumination and eating/grazing behaviour run lengths are shown in Figure 4. A significant Time × Treatment interaction was observed (see Table S4). The behavioural run lengths were similar between the groups across the baseline period, although the AB tended to have greater eating/grazing run lengths. The resting run lengths decreased leading up to separation and remained low for the remainder of the experiment. The fenceline cows had longer resting run lengths on day 2, but the AB cow resting run lengths were 1.5 times longer than those of the FL cows for day 4 to 6. The high-activity run lengths were similar to baseline for all days except day 0 to 2. The abrupt cows had longer high-activity run lengths after separation and when compared to the FL cows from day 0 to 3 and also on day 5. The abrupt cow rumination run lengths were nearly four times longer on days 4 and 5 and two times longer on days 3 and 7. The eating run lengths decreased from baseline after separation and remained lower for the remainder of the experiment. However, the AB cows had greater eating/grazing run lengths on days 4 and 5.

In part 2 separation, both the groups had similar run lengths with minor differences. The resting behaviour run lengths were similar between the groups. High-activity run lengths were similar to baseline and between groups. However, the AB cows had greater high-activity run lengths on day 12 compared to the FL cows. Abrupt cow rumination run lengths were two times longer on day 8, before the FL cows increased rumination run lengths (greater than baseline) to be similar with the AB cows.

**Figure 5:**
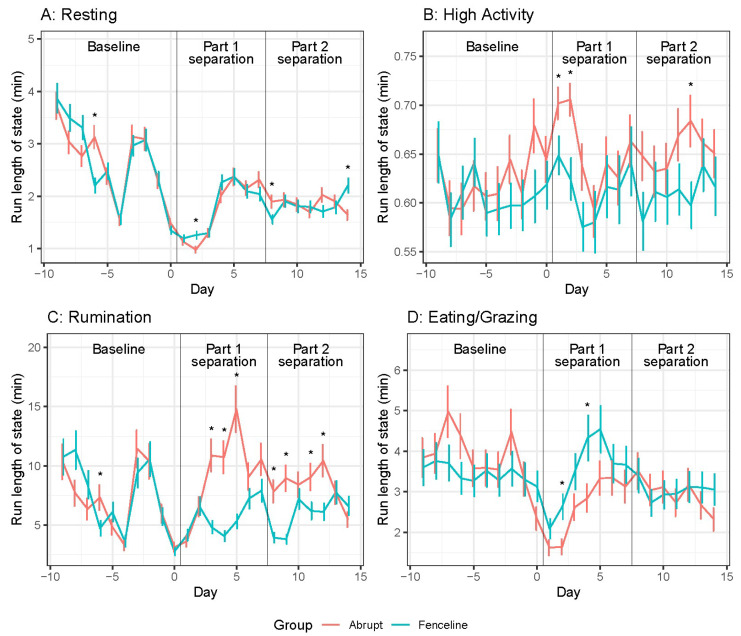
Daily calf behaviour run lengths means ± SE. (**A**) Resting, (**B**) High Activity, (**C**) Rumination, and (**D**) Eating/Grazing. Pairwise significant differences are indicated with an asterisk (*p* < 0.05).

The calf mean resting, high activity, rumination, and eating behaviour run lengths are shown in Figure 5. A significant Time × Treatment interaction was observed (see Table S5). There were similar run lengths during the baseline although the AB calves tended to have greater high-activity and eating/grazing run lengths. The resting run lengths decreased from baseline leading up to separation and continued until day 3 before increasing again. The fenceline calves had greater resting run lengths on day 2 but were similar between the groups for the remainder of separation part 1. During the first 2 d after separation, the AB calves had longer high-activity run lengths compared to the FL calves. Calf rumination was similar across the baseline period and decreased leading up to separation. After separation, the abrupt calves had longer rumination run lengths for 7 out of the 14 d when compared to the FL calves. The eating/grazing run lengths during baseline tended to be greater for the AB calves than the FL calves before decreasing before separation. Both the groups of calves decreased in their eating run lengths by 1.5 times for the first 3 days after separation before increasing back to baseline. However, the FL calves had greater eating run lengths on days 2 and 4 and tended to be greater for the FL calves than the AB calves.

During separation part 2, the resting run lengths were similar, however, the AB calves had greater resting run lengths on day 8 whereas the FL calves had greater run lengths on day 14. On day 12 after separation, the AB calves had longer high-activity run lengths compared to the FL calves. The rumination run lengths were similar to baseline for both the groups, with the FL calves having greater rumination run lengths on days 8 to 12. Eating/grazing was similar between both the groups.

### 3.2. Visual Behavioural Data

**Figure 6:**
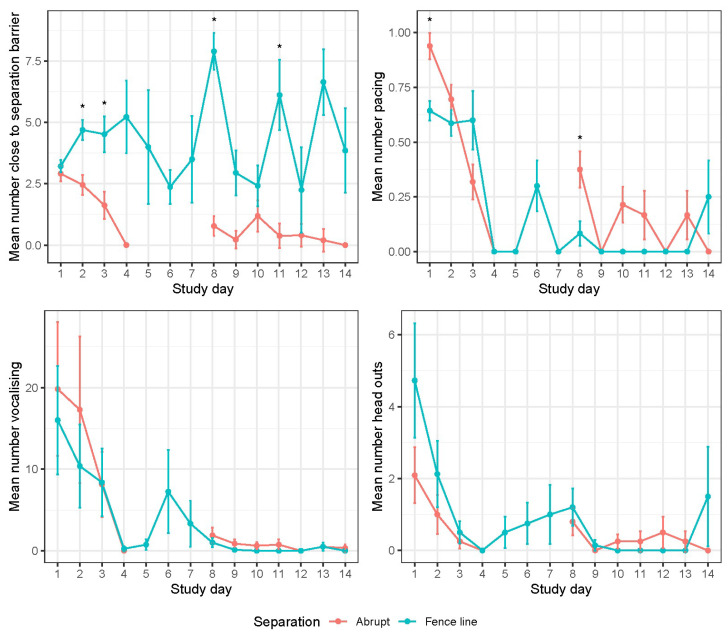
Daily calf visual behaviour frequencies ± SE for close to barrier, pacing, vocalisation, and head outs. Pairwise significant differences are indicated with an asterisk (*p* < 0.05).

**Figure 7:**
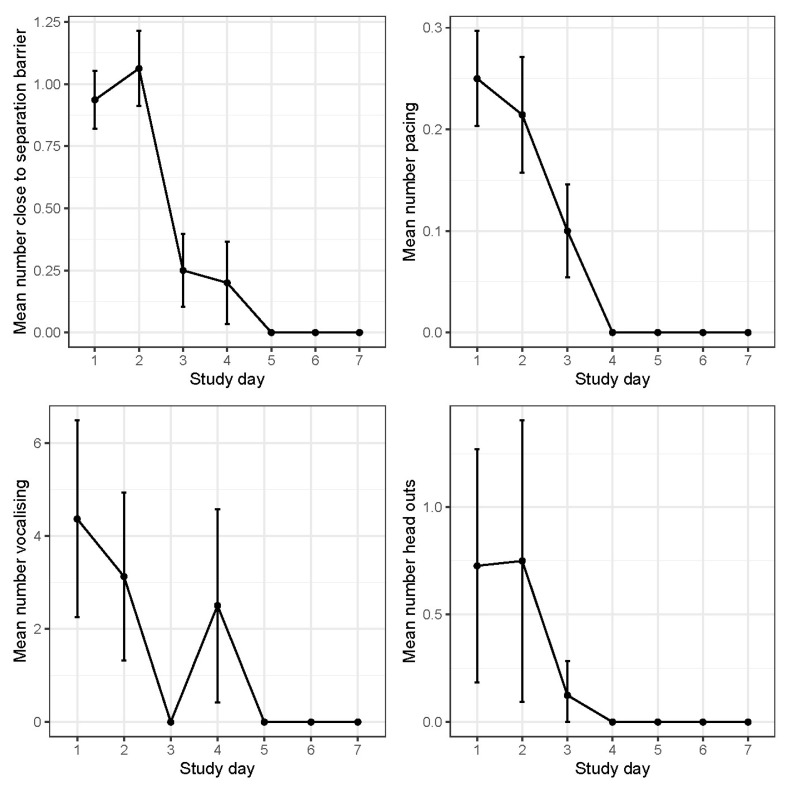
Daily cow visual behaviour frequencies ± SE for close to barrier, pacing, vocalisation, and head outs.

The calf and FL cow video-derived mean behaviours for close to barrier, pacing, vocalisation, and head out are represented in Figure 6 and Figure 7.

#### 3.2.1. Close to Barrier

The fenceline calves were observed close to barrier two times more than the abrupt calves on days 2 and 3 (*p* < 0.05). The rate of occurrence for the FL calves was consistent until day 8 (when cows were removed) where the FL calves peaked in this behaviour (*p* < 0.001). After day 8, the AB calves had a low rate of occurrence near the barrier. The fenceline cows had a low rate of occurrence close to the separation barrier across the entire 7 d recorded. with the greatest occurrence on days 1 and 2. After 2 d, the rate of occurrence decreased 8-fold (*p* < 0.001).

#### 3.2.2. Pacing

Calf pacing had a low occurrence of <1% of the time throughout the experiment although the AB calves had a greater overall occurrence (*p* < 0.001) more specifically on day 1 and 8 (*p* < 0.05). The fenceline calves had similar pacing behaviour for the first 3 days following separation before decreasing to a lower occurrence for the remainder of the experiment. The abrupt calves had the greatest rate of occurrence for pacing on day 1 before slowing declining until day 3. Sporadic increases in AB pacing behaviour were observed on days 8, 10, and 11. The fenceline cows were observed pacing two times more on days 1 and 2 compared to 3 d with an occurrence of >1% for the remaining days.

#### 3.2.3. Vocalisation

Although both the groups had a similar number of vocalisations across all the days, there was a trend for the AB calves to vocalise more than the FL calves (*p* = 0.052). The mean number of calf vocalisations changed over time with a greater number of vocalisations on days 1, 2, and 3, before decreasing in occurrence. However, there was a spike in vocalisations on day 6 (*p* < 0.001). The vocalisation count for the FL cows was the greatest on days 1, 2, and 4 (*p* < 0.001).

#### 3.2.4. Head out

The mean number of calf head outs where similar across the groups; however, there was a day effect with a greater number of occurrences on day 1 before halving on day 2 and continuing to decrease with a slight increase on day 7 and 8 (*p* < 0.001). The frequency of head out behaviour recorded for the FL cows was similar across all days (*p* > 0.05). The fenceline calves were observed attempting to suckle the cows through the fence 37 times across the first 7 d.

#### 3.2.5. Social

The fenceline calves and cows were observed socialising through the fence 91 times (calf initiated: *n* = 47, cow initiated: *n* = 44). Instances of the calves initiating social contact with the cows was greatest the first 3 days of separation before decreasing by half for the remainder of the 7 d. The cows initiating social contact with the calves had the greatest frequencies on days 2, 3, and peaking at 4 d.

### 3.3. Liveweight Change

Mean cow and calf weekly weight, ADG and cortisol data for each group are provided in Table 4 and Table 5.

Calf weight was similar between treatment with a time period effect as they continued to increase across the experiment (*p* < 0.001). During weaning part 1, the ADG of FL calves was nearly three times greater than that AB calves (*p* < 0.001) with a significant decrease in weaning part 2. The ADG of the AB calves was consistent across all stages. Despite the FL calves having a greater ADG in part 1, calf weights were similar across all timepoints.

Cow weights were similar at baseline and increased following weaning with the AB cows having greater mean weights (*p* < 0.05) for subsequent timepoints. The average daily gain of the AB cows was greater than that of the FL cows for both weeks (*p* < 0.05) with the AB decreasing by six times between part 1 and part 2.

### 3.4. Salivary Cortisol

The calves had similar cortisol concentrations for the entirety of the experiment; however, there was a week effect (*p* < 0.001). The greatest mean cortisol concentration was at baseline with a 3-fold decrease at week 1 before increasing at week 2 (*p* < 0.001). However, cow cortisol concentrations were consistent between the groups and timepoints across the entire experiment.

## 4. Discussion

### 4.1. Separation Part 1 Behaviour

The abrupt cows displayed the greatest duration of high activity during the first 2 d following separation. The fenceline cows had a delayed surge of high activity occurring on day 3 suggesting their stress behaviour occurred during this time. When comparing the peaks of high-activity proportions, the abrupt cows had a greater high-activity proportion of time than the FL (in addition to the delay in high activity peaking) suggests that the FL cows were less impacted by separation. High activity can be associated with oestrus [28]; while oestrus could potentially have been responsible for this level of activity, it is unlikely as these cows were pregnant. The sensor categorises high activity as agonistic, play, and stereotypic behaviours. Frustration displayed as a result of cow–calf separation has been documented to cause stereotypic behaviour in previous work [37]. It is likely that a combination of behaviours contributed to these high-activity levels such as pacing post-separation [3,38] as recorded by video in the current experiment. Even though the FL cows had less of a high-activity surge during the first 2 d, they were still impacted by separation. Due to the limited contact to their calves and observed suckling attempts in the video, this may cause stress and frustration [8,37]. As the FL cows had a delayed surge of high-activity stress response on 3 d, this suggests that a shorter period of two-step weaning using a fenceline could prevent this frustration.

The fenceline cows had a greater duration of resting time than the AB cows for the first 7 days of the experiment. A decrease in resting time has been correlated to high stress responses [10], suggesting that the AB cows had a greater stress response when compared to the FL cows. However, the AB cows had greater resting run lengths on days 4 to 6, suggesting less undisturbed resting bouts. Although resting was not measured in the video data, the FL cows were seen resting in the pen directly next to the calves, especially at night. Although it should be noted that in the sensor data resting time does not equate to lying time (lying time is part of resting behaviour but not exclusively), the only comparable measure from the previous literature is lying time. However, our results contrast with the previous literature that reported no difference in lying time between AB and FL cows [3,38]. In the current experiment, behaviour was measured using technology at short time intervals (min by min) compared to interval samples of 3 h [3] or 4 h [38] blocks in the previous work. Technology allows for more frequent and objective recording intervals to provide a more accurate behavioural time budget to assess the impact and motivation of cows around calf weaning. Although an increase in resting time suggests less of a stress response, too much resting time starts to impact other behaviours necessary for maintaining cows’ body condition.

Abrupt cow eating times were similar to baseline throughout the experiment but had greater eating times when compared to those of the FL cows from day 3 to 7. Abrupt cow eating time run lengths were greater than those of the FL cows on days 3 and 4, suggesting less interrupted eating times. Our results contradict the previous research reporting cows whose calves were abruptly weaned grazed less after separation compared to those whose calves were weaned using a nose flap [3,38]. Although decreased eating times have been associated with stress responses, an explanation for this decrease could be the greater resting times for the FL cows in the pen next to the calves. This is not reflected in the close-to-separation-barrier visual behaviour; however, the definition of this behaviour is within 2 m from the fenceline, and the cows resting outside of this range would not be recorded. Due to the decrease in eating time for the FL cows after day 3, a shorter duration of fenceline separation before full separation could prevent this decrease in eating time. The cows increased their rumination time after separation, contrasting the previous work showing AB cows’ decreased rumination time after separation [38]. However, total rumination time for the FL cows was lower than that of the AB cows on day 2, although the rumination time remained similar for both the groups following day 3 until day 7 where the FL cows had another decrease in rumination time. A decrease in rumination has been reported as a stress response [39,40]. Ungerfeld et al. [3] recorded no difference in cow rumination time between abrupt and two-step separated groups. It is likely the decrease in rumination on these days can be attributed to stress around the transition time of partial separation (day 2) and full separation (days 7 and 8). In this regard, the FL treatment had a greater impact on cow eating and rumination behaviour when they had limited contact with their calves during the first week. Implementing full separation after 3 days would improve and prevent the second decrease in rumination.

During part 1 separation, the FL calves ruminated and rested more than the AB calves. As rumination is a positive welfare indicator [41,42], the FL-weaned calves had a reduced stress response to weaning, in line with the previous research reporting increased lying and rumination time of two-step weaned calves when compared to abruptly weaned calves [9,13,43]. However, others have reported no differences in lying time when comparing nose flaps to abrupt weaning [8,10]. Boland et al. [44] recorded greater lying times for calves weaned with nose flaps but no difference between AB calves and FL calves. A decrease in lying time is an indicator of negative welfare in cattle [1]. Abrupt calves were observed pacing more than FL calves specifically during the first 3 d following separation and is in line with the previous studies reporting AB calves to spend a greater time walking [9,10,44]. The fenceline calves had greater rumination durations in the first 2 d following separation as compared to the AB calves but their rumination levels were similar between treatments for the remainder of the experiment. Our results are in line with Loberg et al. [9], where two-step weaned calves ruminated more the first few days following separation. Greater rumination during the first 2 days suggests the FL calves were less stressed than the AB calves. Conversely, abruptly weaned calves have also been reported to have greater rumination times [8] and Boland et al. [44], reported no difference between two-step or abruptly weaned calf rumination. The calves rebound quickly to the initial stress of weaning (within the first 2 d), suggesting the benefits of limited contact with the cows through a fenceline occurred during the first few days, and it may be unnecessary to maintain contact for longer periods of time before full separation.

Abruptly weaned calves had greater high-activity times during the first 3 d compared to the FL calves, indicating a greater stress response. Although technology can be used for oestrus detection by monitoring mounting, no mounting behaviour was observed in the calves in the current work. Similar to cows, frustration resulting from cow–calf separation has been documented to cause stereotypic behaviour in calves reported by the previous work [37]. Nonetheless, greater occurrences of pacing, seeking behaviour, and a decrease in recumbent behaviours has been reported as indicators of stress around separation [45]. More consistent indicators of distress are the presence and increase in vocalisation [8,46]. Even though both calf groups had similar numbers of vocalisations across the experiment, the high occurrence of this behaviour during the first 3 d suggests calves experienced similar distress. Conversely, the previous research reported that two-step weaned calves vocalised four times more than abruptly weaned calves [43], with abruptly weaned calves vocalising more [9] during the day following separation; however, similar to our results, both groups returned to baseline by day 3 [9] or 4 [43]. As the sensor and visually observed behaviour suggests, the first 3 d were the most stressful and the FL calves were less impacted than the AB calves, leading to the recommendation of limiting cow–calf contact to the first 3 d.

### 4.2. Separation Part 2 Behaviour

A second spike in high activity was recorded for the AB cows on day 8, although this was for a smaller proportion of time than day 1. This second peak of high activity could be due to the social stresses of introducing the FL cows to their established social order. Agonistic behavioural responses have been observed during the first 3 d of regrouping of cattle as a result of feed competition and establishment of the dominance hierarchy [47,48]. The second surge of high activity could have been prevented if the FL cows were fully separated from their calves within the first 3 days (where the first peak of high activity was recorded), resulting in one peak of high activity. The lack of stress response from the FL cows after part 2 of separation could be due to the decrease in the frequency of socialisation and close-to-barrier behaviours during the end of part 1 separation, suggesting habituation to the separation. Both socialisation and close-to-barrier behaviours were negligible after day 4 following separation. The cows with their calves abruptly weaned seemed to have a greater stress behavioural response when compared to the FL cows; however, there is a paucity in observations of cow behaviour around weaning.

During part 2 separation, the FL cows’ resting time decreased directly after full separation, while AB cows’ resting times were consistent. The decrease in resting time is likely due to the absence of their calves and the cows spending more time eating. Significant drops in rumination times for the FL cows can be seen on day 2, 7, and 8. A decrease in rumination has been reported as a stress response [39,40]. Ungerfeld et al. [3] recorded no difference in rumination time between the groups during the second week of observations. Stress in the fenceline cows is a result of limited contact with their calves at day 1 and complete separation at day 8 thereafter. There seems to be a greater impact to FL cows’ rumination time directly after each step of separation. To minimise the impact around full separation, FL cows should be fully separated from their calves within the first 3 days which would result in one stress event instead of two.

For the majority of part 2 separation, the calves had similar resting, high-activity, rumination and eating behaviour. However, the AB calves had a second increase in high activity on days 6 to 8 which is unexplainable. Nonetheless, these similarities in behaviour between groups, suggest the FL calves were not impacted by the second step of fenceline weaning. If FL calves were not impacted by full separation, this event could potentially occur earlier than 7 d.

### 4.3. Weight

In our experiment, both the FL and AB cows continued to gain weight after weaning although the AB cows had greater BW on days 7 and 14 when compared to the FL cows. Cattle tend to decrease in weight gain as a result of stress [39]. Although both the cow groups continued to increase in BW, the FL cows’ decreased eating behaviour during the first week could explain the divergence in BW for each group. These results contradict the previous work reporting similar BW for both AB and FL cows [3,11]. The ADG during part 1 separation was six times greater in the AB cows than the FL cows, with the AB cows decreasing in ADG whereas the FL cows increased in ADG during part 2 separation. It is clear, during part 1 separation, that the FL cows were impacted enough to affect weight gain in the long term. To decrease the negative impact on FL cows’ weight during part 1 separation, implementing full separation earlier within the first week could be undertaken.

The fenceline calves had greater ADG than the AB calves during part 1 separation with a severe decrease in ADG for the FL calves in part 2 separation. The abrupt calves had similar ADG during part 1 and part 2 separation. However, both the groups had similar BW across the entire experiment. Potentially, some of the growth of the FL calves during part 1 separation could be attributed to suckling through the fence which was occasionally visually observed. The previous literature comparing calf growth rates between two-step to abrupt weaning is inconclusive [8,11,44]. Our results during part 1 separation were in line with the previous work reporting ADG of FL calves to be greater than that of AB calves [13,14] but contrasted with several studies reporting similar ADG after the first week post-weaning [9,10,11]. It is common for beef cattle to gain less weight during stressful situations [39], which could be reflected in the FL ADG during part 2 separation. Enríquez et al. [8] reported AB calves to have greater overall ADG than FL calves. Similar to our results, 6-month-old calves weaned AB and FL had similar ADG during the second week post-separation [8], whereas others report 6- to 8-month-old FL calves with greater ADG [13] when compared to AB weaning. The contradicting ADG results suggest an external factor other than the weaning method could be affecting weight gain. Fully separating FL cows and calves during the part 1 separation, could have prevented the 4-fold decrease in ADG in the FL calves during part 2 separation. The inconsistency suggests external factors affecting calf weight gain, and further work is necessary to assess other aspects that could be affecting calf weight gain.

### 4.4. Cortisol Concentrations

Cow cortisol concentrations were maintained at baseline levels for both groups at all the sample times. The research on cow cortisol concentrations in response to weaning is limited with the focus primarily being on the response of calves, despite the motivation of weaning for farmers being centred around maintaining the reproductive and physical health of their cows [3,4,5]. An increase in cow cortisol concentrations has been reported in response to high-stress situations such as separation from the herd, novel environments, entering squeeze chutes, and blood collection [39] and has been reported to decrease progesterone and contribute to the termination of pregnancy [49]. Ewes have experienced anoestrous in response to increased cortisol concentrations [50]. As no long-term cortisol impact was observed, it is unlikely these cows would face repercussions on future reproduction.

Cortisol concentrations were similar across all timepoints for the FL and AB calves suggesting both the groups of calves had similar physiological stress responses to weaning. Our results are in line with the previous research reporting similar plasma cortisol concentrations when comparing calves weaned abruptly, with nose flaps [11,15], or by fencelines [15]. Although their saliva cortisol concentrations are lower than their plasma levels [51], there is a correlation between saliva and plasma cortisol samples in cattle [51,52] that allows us to have a non-invasive [53] way to observe cortisol trends. However, the previous research reported calves weaned using a two-step weaning method had lower faecal cortisol concentrations in the first few days following weaning when compared to that following abrupt weaning [11,16,54]. As only weekly samples were collected, it is likely these acute changes in cortisol concentrations during the first few days of cow–calf removal were not captured. The greatest cortisol concentrations for calves were at baseline with a 4-fold decrease at 7 d and then increasing slightly at 14 d post-weaning. This peak at baseline is most likely due to the lack of habituation to the yards and their being drafted from their mothers for the first time. The previous research reported higher cortisol concentrations due to the stress of handling and restraint [55] and has been reported to potentially confound measurements [56]. A decrease from baseline 7 d following weaning has been recorded in an evaluation of the impact of the presence of pre-weaning nose flaps on calf physiology and performance when weaned through fencelines and abrupt weaning [15]. Conversely, a study comparing dairy calves weaned with nose flaps and abruptly reported a decrease in cortisol concentrations from baseline for two-step weaning during the first 4 d following separation while abruptly weaned calves increased in cortisol concentrations [9]. Further research involving more frequent sampling is necessary to interpret the decrease in cortisol at day 7.

## 5. Conclusions

The effects of abrupt and fenceline weaning on cow and calf behaviour, saliva cortisol concentrations, and weight gain were compared over a 14 d period. The use of a sensor ear tag allowed for continuous, objective behaviour data collection. Although both the groups displayed stress behaviours, all occurrences of stress in the FL cows could potentially be mitigated or decreased further if they were fully separated at 3 days post-weaning. The abruptly separated cows were more stressed at weaning when compared to the FL cows due to the longer and more intense high-activity behaviour and increased rest of AB cows. However, the FL cows were still impacted by stress; lower rumination run lengths and eating behaviour were recorded starting on day 4, resulting in the lower weight gains. These impacts could be mitigated if cows and calves were maintained by a fenceline for 3 days and then fully separated. Although the FL calves also had a stress response, it was less than that of the AB calves and resolved after the first 2 d after weaning with an increase in recumbent behaviours. Overall, abrupt weaning is more stressful for both the cows and calves when compared to fenceline weaning. Together, these results suggest that calves be fenceline-weaned for 3 days followed by total separation. However, further work is required to test this hypothesis and determine if the recommendation holds for other breeds and environments.

## Figures and Tables

**Table 1:** Description of behaviours measured by the triaxial sensor ear tag.

Behaviour	Description
Resting	Standing still, lying, and transition between these two events. While lying, allowed to do any kind of movement with head/neck/legs (e.g., tongue rolling).
Rumination	Rhythmic circular/side to side movements of jaw not associated with eating or medium activity, interrupted by brief pauses (<5 s) during time that bolus is swallowed and then regurgitated, followed by continuation of rhythmic jaw movements.
High activity	Includes any combination of running, mounting, head-butting, repetitive head-weaving/tossing, leaping, buck-kicking, and rearing.
Eating/grazing	Muzzle/tongue physically comes into contact with feed and manipulates it, often but not always followed by visible chewing. Can include grazing while either standing in place or moving at slow, even or uneven pace between patches.

**Table 2:** Ethogram with description of cow and calf behaviours measured during visual observations categorised by focal animal and weaning group modified from Loberg et al. [9] and Enríquez et al. [8].

Behaviour	Focal Animal	Group	Type	Description
Vocalisation ^a^	Cow, Calf	AB, FL	Point	Compression of the calf’s diaphragm, elongation of the neck, with either an open or closed mouth.
Head out	Cow, Calf	AB, FL	Point	Standing with nose and/or head outside the pen at the separation barrier with the eyes and ears focused in the same direction
Social	Cow, Calf	FL	Point	Initiating sniffing, licking, or rubbing between cows and calves
Suckling Attempt	Calf	FL	Point	Rewarded or non-rewarded suckling attempt. Head through separation barrier when dam is close, nuzzling of udder, and/or teat enclosed in mouth
Close to separation barrier ^1^	Cow, Calf	AB, FL	Point, Count	Positioned so that any part of the head is within 2 m of the separation barrier
Pacing ^1^	Cow, Calf	AB, FL	Point, Count	A minimum of two steps moving parallel to and within 2 m of the separation barrier

^a^ Modified from [29]; ^1^ Documenting number of cows or calves displaying the behaviour with a new measurement anytime that number changes.

**Table 3:** Video analysis timepoints given in hours for each day of the experiment observed in 15 min block intervals.

Day	Timepoints ^1^
1 and 8	0, 1, 2, 3, 6, 9, 12, 15, 18, 21, 24
2, 3, 9, and 10	3, 6, 9, 12, 15, 18, 21, 24
4 to 7 and 11 to 14	6, 12, 18, 24

^1^ Timepoints are displayed as hours with timepoint 0 representing when calves first entered their respective pens.

**Table 4:** Mean cow weekly weights, average daily gain, and cortisol concentrations for fenceline and abrupt weaning treatments.

				*p* Value		
	Time Period	Abrupt	Fenceline	Time Period	Treatment	Time Period × Treatment
Weight	Day 0	363 ± 9.7	353 ± 9.7	2.7 × 10^−12^	0.03	0.01
	Day 7	407 ± 9.7	366 ± 10.1			
	Day 14	421 ± 9.8	387 ± 9.7			
ADG	Part 1 ^a^	6.21 ± 0.99	1.91 ± 1.06	4.5 × 10^−3^	0.02	5.1 × 10^−8^
	Part 2 ^b^	2.05 ± 1.01	3.0 ± 1.07			
Cortisol	Day 0	3.6 ± 5.2	16.7 ± 5.2	0.42	0.13	0.42
	Day 7	3.5 ± 5.5	11.5 ± 5.3			
	Day 14	3.5 ± 5.3	4.8 ± 5.5			

^a^ Day 0 to 7, ^b^ Day 7 to 14. Average daily gain (ADG).

**Table 5:** Mean calf weekly weights, average daily gain, and cortisol concentration for fenceline and abrupt weaning treatments.

				*p* Value		
	Time Period	Abrupt	Fenceline	Time Period	Treatment	Time Period × Treatment
Weight	Day 0	150 ± 6.8	149 ± 6.8	8.8 × 10^−5^	0.71	0.04
	Day 7	159 ± 6.8	178 ± 6.8			
	Day 14	169 ± 6.8	181 ± 6.8			
ADG	Part 1 ^a^	1.3 ± 0.5	4.2 ± 0.5	0.61	0.15	0.002
	Part 2 ^b^	1.4 ± 0.6	0.4 ± 0.5			
Cortisol	Day 0	4.4 ± 0.6	5.2 ± 0.6	2.4 × 10^−6^	0.82	0.22
	Day 7	1.9 ± 0.6	1.6 ± 0.6			
	Day 14	3.9 ± 0.6	3.0 ± 0.6			

^a^ Day 0 to 7, ^b^ Day 7 to 14. Average daily gain (ADG).

## Data Availability

The data presented in this experiment are available on request from the corresponding author. The data are not publicly available due to third-party ownership of some of the data.

## Supplementary Materials

The following supporting information can be downloaded at: https://www.mdpi.com/article/10.3390/ani14111525/s1. Appendix S1: University of Sydney bovine saliva collection standard operating procedure; Table S1: *p*-values, SD, and 95% CI for cow behaviour proportions; Table S2: *p*-values, SD, and 95% CI for calf behaviour proportions; Table S3: *p*-values, SD, and 95% CI for cow and calf behaviour diversity; Table S4: *p*-values, SD, and 95% CI for cow behaviour run lengths; Table S5: *p*-values, SD, and 95% CI for calf behaviour run lengths.
