# Supplementary material for: The Impact of Abrupt and Fenceline-Weaning Methods on Cattle Stress Response, Live Weight Gain, and Behaviour
Source: Animals (Basel). 2024 May 22;14(11):1525. doi: 10.3390/ani14111525 (PMC11171169; doi:10.3390/ani14111525)
Supplement: Supplementary file 1 [file animals-14-01525-s001.zip › Appendix SA.pdf]

THE UNIVERSITY OF SYDNEY

ANIMAL ETHICS COMMITTEE
STANDARD OPERATING PROCEDURE

**SOP Title:**	**Collection of Saliva**		
**Species:**	**Bovine**		
**Faculty/School:**	Science/SOLES		**Initial issue date: 01.03.19**<br>**Next review date:**
**AEC SOP number:**		**Version: 1**	**Version issue date:**
**Author:**	Sabrina Lomax		
**Circulation*:**	Can be made available to other researcher groups (via IRMA / intranet)		

*(* Circulation – 'Drop down list', Please indicate if this SOP can be made available to other investigators within the University of Sydney)*

Summary

This Standard Operating Procedure (SOP) describes the procedure of collection of a sample of parotid saliva from restrained animals in order to measure the levels of various physiologically important substances, in particular, steroid hormones (eg free cortisol), sodium and potassium, vanillyl mandelic acid etc.

Scope

This procedure is to be applied by all investigators who are trained and deemed competent in the procedure of saliva sampling from calves and cows.

Staff or students not trained and deemed competent in this procedure should not perform this procedure.

Compliance and Competence Requirements

Supervisors are responsible for ensuring that all investigators authorised to use this SOP have achieved an acceptable level of understanding and competence in the procedure.

Approved investigators are responsible for following all instructions covered in this SOP and for using equipment provided for personal protection and animal welfare purposes.

Each animal will be observed and monitored according to the *Australian code for the care and use of animals for scientific purposes 8th Edition 2013* and the approved project.

Animal Health and Welfare Considerations

The method is non-invasive and thus has little effect on the well-being of the animal.

Potential adverse impact	Control measures
Distress from restraint	Quiet handling, gentle restraint in crush, minimize time for procedure

Potential Hazards / Safety Precautions for Personnel

(List any potential hazards / safety precautions that may need to be considered by personnel working with the animals, disposing of animal waste and conducting the initial procedure. Please include the control measures used to reduce the likelihood and severity of those hazards. If required, please complete a risk assessment found on the Safety Health & Wellbeing webpage.)

Potential Hazard	Control measures
Q-fever	All staff should be vaccinated or wear PPE
Handling large animals – head	Use proper restraint in head bail, apply chin bar to minimize movement of the head

(These factors should be addressed when completing risk assessments for projects using this procedure)

All incidents must be reported via RiskWare within 24 hours.

Equipment and resources

(Including personal protective clothing (PPE), chemicals and equipment needed)

PPE

- If not Q-fever vaccinated must wear protective clothing (overalls), gloves, P2 mask + glasses

Equipment

- Gloves, sterile pipette, sample tube

Procedure

(Provide a step-by-step description of the procedure/technique. Diagrams and photos may be of assistance.)

The animal is restrained with conventional restraint techniques (eg held in a cattle crush) and head movement is restrained, either by using the chin bar on the crush held by a person. A soft plastic bulb pipette with a rubber bulb, a vacuum-operated pipette or other aspiration device with a plastic disposable pipette, is inserted between the cheek and the lower jaw, along the side of the mouth towards the back teeth. Saliva is drawn into the pipette, either by the bulb or the aspiration device. The pipette is withdrawn and the sample placed into a collection tube.

References and Acknowledgements

https://www.dpi.nsw.gov.au/animals-and-livestock/animal-welfare/general/general-welfare-of-livestock/sop/cattle/health/saliva-collection
