# Supplementary material for: The Impact of Abrupt and Fenceline-Weaning Methods on Cattle Stress Response, Live Weight Gain, and Behaviour
Source: Animals (Basel). 2024 May 22;14(11):1525. doi: 10.3390/ani14111525 (PMC11171169; doi:10.3390/ani14111525)
Supplement: Supplementary file 1 [file animals-14-01525-s001.zip › Table S1.pdf]

Table S1. Significance levels of terms (P-values), and standard deviation with 95% confidence interval for cow random effects, for each behaviour proportion of time detected from the sensor ear tag for cows separated from their calf abruptly or by a fenceline.

	P-value			
Behaviour	Day	Treatment	Day × Treatment	Cow SD (95% CI)
Resting	$< 2\times10^{-16**}$	0.0018^{*}	$< 2\times10^{-16**}$	0.164 (0.122, 0.214)
High Activity	$< 2\times10^{-16**}$	0.17	0.0051^{*}	0.284 (0.207, 0.373)
Rumination	$< 2\times10^{-16**}$	0.50	0.027^{*}	0.384 (0.285, 0.499)
Eating and Grazing	$< 2\times10^{-16**}$	0.10	$< 2\times10^{-16**}$	0.152 (0.113, 0.198)

$^{*}P < 0.05$, $^{**}P < 0.001$
