# Supplementary material for: The Impact of Abrupt and Fenceline-Weaning Methods on Cattle Stress Response, Live Weight Gain, and Behaviour
Source: Animals (Basel). 2024 May 22;14(11):1525. doi: 10.3390/ani14111525 (PMC11171169; doi:10.3390/ani14111525)
Supplement: Supplementary file 1 [file animals-14-01525-s001.zip › Table S2.pdf]

Table S2. Significance levels of terms (P-values), and standard deviation with 95% confidence interval for calf random effects, for each behaviour proportion of time detected from the sensor ear tag for calves weaned abruptly or by a fenceline.

	P-value			
Behaviour	Day	Treatment	Day × Treatment	Calf SD (95% CI)
Resting	$< 2\times10^{-16**}$	0.37	$3.8\times10^{-8**}$	0.144 (0.108, 0.186)
High Activity	$< 2\times10^{-16**}$	0.052	$2.0\times10^{-7**}$	0.511 (0.387, 0.656)
Rumination	$< 2\times10^{-16**}$	0.15	$2.8\times10^{-7**}$	0.089 (0.065, 0.117)
Eating and Grazing	$< 2\times10^{-16**}$	0.85	0.014^{*}	0.397 (0.304, 0.506)

$^{*}P < 0.05$, $^{**}P < 0.001$
