# Supplementary material for: The Impact of Abrupt and Fenceline-Weaning Methods on Cattle Stress Response, Live Weight Gain, and Behaviour
Source: Animals (Basel). 2024 May 22;14(11):1525. doi: 10.3390/ani14111525 (PMC11171169; doi:10.3390/ani14111525)
Supplement: Supplementary file 1 [file animals-14-01525-s001.zip › Table S3.pdf]

Table S3. Significance levels of terms (P-values), and standard deviation with 95% confidence interval for cow and calf random effects, for behavioural diversity of cows and calves separated abruptly and by fenceline.

	P-value			
Cow/Calf	Day	Treatment	Day × Treatment	Cow/Calf SD (95% CI)
Cows	$< 2\times10^{-16**}$	0.95	$6.0\times10^{-5**}$	0.095 (0.072, 0.122)
Calves	$< 2\times10^{-16**}$	0.62	$8.1\times10^{-8**}$	0.073 (0.055, 0.093)

$^{*}P < 0.05$, $^{**}P < 0.001$
