# Supplementary material for: The Impact of Abrupt and Fenceline-Weaning Methods on Cattle Stress Response, Live Weight Gain, and Behaviour
Source: Animals (Basel). 2024 May 22;14(11):1525. doi: 10.3390/ani14111525 (PMC11171169; doi:10.3390/ani14111525)
Supplement: Supplementary file 1 [file animals-14-01525-s001.zip › Table S4.pdf]

Table S4. Significance levels of terms (P-values), and standard deviation with 95% confidence interval for cow random effects, for each behaviour run length detected from the sensor ear tag for cows separated from their calf abruptly or by a fenceline.

	P-value			
Behaviour	Day	Treatment	Day × Treatment	Cow SD (95% CI)
Resting	$< 2\times10^{-16**}$	0.16	$2.6\times10^{-15**}$	0.165 (0.122, 0.214)
High Activity	0.000427^{**}	0.093	0.040^{*}	0.03 (0.012, 0.045)
Rumination	$< 2\times10^{-16**}$	0.0071^{*}	$< 2\times10^{-16**}$	0.173 (0.120, 0.231)
Eating and Grazing	$< 2\times10^{-16**}$	0.56	$< 2\times10^{-16**}$	0.454 (0.346, 0.579)
All states	$< 2\times10^{-16**}$	0.70	$< 2\times10^{-16**}$	0.145 (0.110, 0.186)

$^{*}P < 0.05$, $^{**}P < 0.001$
