# Supplementary material for: The Impact of Abrupt and Fenceline-Weaning Methods on Cattle Stress Response, Live Weight Gain, and Behaviour
Source: Animals (Basel). 2024 May 22;14(11):1525. doi: 10.3390/ani14111525 (PMC11171169; doi:10.3390/ani14111525)
Supplement: Supplementary file 1 [file animals-14-01525-s001.zip › Table S5.pdf]

Table S5. Significance levels of terms (P-values), and standard deviation with 95% confidence interval for calf random effects, for each behaviour run length detected from the sensor ear tag for calves weaned abruptly or by a fenceline.

	P-value			
Behaviour	Day	Treatment	Day × Treatment	Calf SD (95% CI)
Resting	$< 2\times10^{-16**}$	0.95	$1.0\times10^{-9**}$	0.138 (0.103, 0.178)
High Activity	0.00010^{**}	0.052	0.73	0.058 (0.040, 0.077)
Rumination	$< 2\times10^{-16**}$	0.057	$< 2\times10^{-16**}$	0.302 (0.228, 0.387)
Eating and Grazing	$< 2\times10^{-16**}$	0.67	$4.5\times10^{-16**}$	0.401 (0.306, 0.510)
All states	$< 2\times10^{-16**}$	0.48	$< 2\times10^{-16**}$	0.134 (0.102, 0.171)

$^{**}P < 0.001$
